# Posicionamento sobre Segurança Cardiovascular das Vacinas contra COVID-19 - 2022

**DOI:** 10.36660/abc.20220179

**Published:** 2022-04-07

**Authors:** Humberto Graner Moreira, Múcio Tavares de Oliveira, Bruno Pereira Valdigem, Cristiane Nunes Martins, Carisi Anne Polanczyk

**Affiliations:** 1 Liga de Hipertensão Arterial Universidade Federal de Goiás Goiânia GO Brasil Liga de Hipertensão Arterial - Universidade Federal de Goiás, Goiânia, GO – Brasil; 2 Hospital Israelita Albert Einstein São Paulo SP Brasil Hospital Israelita Albert Einstein, São Paulo, SP – Brasil; 3 Centro de Infusão InCor São Paulo SP Brasil Centro de Infusão e Hospital Dia do InCor, São Paulo, SP – Brasil; 4 Faculdade de Medicina Universidade de São Paulo São Paulo SP Brasil Faculdade de Medicina da Universidade de São Paulo (FMUSP), São Paulo, SP – Brasil; 5 DEIC Rio de Janeiro, RJ Brasil Departamento de Insuficiência Cardíaca da Sociedade Brasileira de Cardiologia (DEIC) - Diretoria 2022-2023, Rio de Janeiro, RJ – Brasil; 6 SOCESP Rio de Janeiro, RJ Brasil Projeto Insuficiência Cardíaca da Sociedade de Cardiologia do Estado de São Paulo (SOCESP),Rio de Janeiro, RJ – Brasil; 7 Rede D’Or Rio de Janeiro, RJ Brasil Rede D’Or, Rio de Janeiro, RJ – Brasil; 8 Instituto Dante Pazzanese de Cardiologia Rio de Janeiro, RJ Brasil Instituto Dante Pazzanese de Cardiologia,Rio de Janeiro, RJ – Brasil; 9 Biocor Instituto Nova Lima MG Brasil Biocor Instituto, Nova Lima, MG – Brasil; 10 Programa de Pós-graduação em Cardiologia Universidade Federal do Rio Grande do Sul Porto Alegre RS Brasil Programa de Pós-graduação em Cardiologia da Universidade Federal do Rio Grande do Sul, Porto Alegre, RS – Brasil; 11 Hospital de Clínicas de Porto Alegre Porto Alegre RS Brasil Hospital de Clínicas de Porto Alegre, Porto Alegre, RS – Brasil; 12 Instituto Nacional de Avaliação de Tecnologia em Saúde Porto Alegre RS Brasil Instituto Nacional de Avaliação de Tecnologia em Saúde, Porto Alegre, RS – Brasil; 13 Hospital Moinhos de Vento Porto Alegre RS Brasil Hospital Moinhos de Vento, Porto Alegre, RS – Brasil

**Table:** 

Posicionamento sobre Segurança Cardiovascular das Vacinas contra COVID-19 – 2022	
O relatório abaixo lista as declarações de interesse conforme relatadas à SBC pelos especialistas durante o período de desenvolvimento deste posicionamento, 2022.	
Especialista	Tipo de relacionamento com a indústria
Bruno Pereira Valdigem	Nada a ser declarado
Carisi Anne Polanczyk	Declaração financeira A - Pagamento de qualquer espécie e desde que economicamente apreciáveis, feitos a (i) você, (ii) ao seu cônjuge/ companheiro ou a qualquer outro membro que resida com você, (iii) a qualquer pessoa jurídica em que qualquer destes seja controlador, sócio, acionista ou participante, de forma direta ou indireta, recebimento por palestras, aulas, atuação como proctor de treinamentos, remunerações, honorários pagos por participações em conselhos consultivos, de investigadores, ou outros comitês, etc. Provenientes da indústria farmacêutica, de órteses, próteses, equipamentos e implantes, brasileiras ou estrangeiras: - Bayer, Pfizer, Novartis, Roche, Amgen, Bristol. Outros relacionamentos Financiamento de atividades de educação médica continuada, incluindo viagens, hospedagens e inscrições para congressos e cursos, provenientes da indústria farmacêutica, de órteses, próteses, equipamentos e implantes, brasileiras ou estrangeiras: - Bayer, Roche.
Cristiane Nunes Martins	Nada a ser declarado
Humberto Graner Moreira	Declaração financeira A - Pagamento de qualquer espécie e desde que economicamente apreciáveis, feitos a (i) você, (ii) ao seu cônjuge/ companheiro ou a qualquer outro membro que resida com você, (iii) a qualquer pessoa jurídica em que qualquer destes seja controlador, sócio, acionista ou participante, de forma direta ou indireta, recebimento por palestras, aulas, atuação como proctor de treinamentos, remunerações, honorários pagos por participações em conselhos consultivos, de investigadores, ou outros comitês, etc. Provenientes da indústria farmacêutica, de órteses, próteses, equipamentos e implantes, brasileiras ou estrangeiras: - Novartis: Entresto; Bayer: Xarelto; Pfizer: Eliquis; Libbs: Plenance Eze.
Mucio Tavares de Oliveira Júnior	Declaração financeira B - Financiamento de pesquisas sob sua responsabilidade direta/pessoal (direcionado ao departamento ou instituição) provenientes da indústria farmacêutica, de órteses, próteses, equipamentos e implantes, brasileiras ou estrangeiras: - Sanofi Pasteur: FLUZONE Senior; Torrent: droga experiental. Outros relacionamentos Financiamento de atividades de educação médica continuada, incluindo viagens, hospedagens e inscrições para congressos e cursos, provenientes da indústria farmacêutica, de órteses, próteses, equipamentos e implantes, brasileiras ou estrangeiras: - Astra Zeneca, Boehringer, Novartis, Torrent Pharma, Sanofi Pasteur, Merck, Biolab.

**Figura Central:**
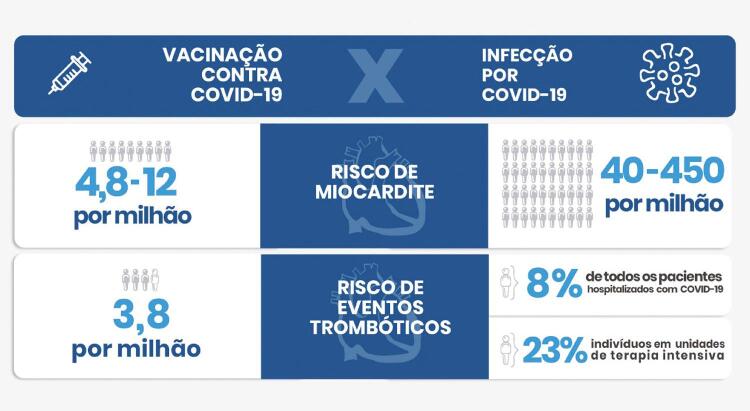
: Posicionamento sobre Segurança Cardiovascular das Vacinas contra COVID-19 - 2022

Arq Bras Cardiol. 2022; 118(4):789-796

## Introdução

O Comitê Científico da Sociedade Brasileira de Cardiologia, por determinação do seu Conselho Administrativo, convocou em grupo de trabalho para monitorar e organizar, de forma continuada e sistemática, evidências científicas da segurança cardiovascular das vacinas contra COVID-19. O objetivo desse grupo de trabalho é reproduzir dados cientificamente sólidos, sintetizar a evidência no momento disponível, e oferecer recomendações para o cardiologista brasileiro na forma de posicionamentos da SBC.

As vacinas para prevenir a infecção por SARS-CoV-2 são consideradas a abordagem mais efetiva para controlar a pandemia pelo vírus. Apesar dos tempos exíguos do desenvolvimento das vacinas contra COVID-19, cada vacina aprovada passou por todas as fases pré-clínicas e clínicas (fases I a III) de pesquisa científica.

Os mesmos critérios rigorosos de segurança aos quais esses estudos foram submetidos permanecem ativos e vigilantes na chamada “fase IV”, ou monitoramento pós-comercialização. Esta fase é fundamental para avaliar a ocorrência de eventos adversos raros que tenham relação causal com as vacinas, pois eles só ficam evidentes com a aplicação em grande número de indivíduos.

Como em outras vacinas, eventos adversos foram observados durante esta fase de monitoramento dos programas de imunização populacional contra COVID-19, alguns relacionados ao envolvimento do aparelho circulatório. A seguir, são revisadas as evidências de dois desses efeitos adversos de interesse quanto à saúde cardiovascular: trombose com trombocitopenia e miocardite induzida pela vacina.

### Trombose com trombocitopenia imune induzida por vacina (VITT)

Em fevereiro de 2021, uma síndrome pró-trombótica foi descrita em um pequeno número de indivíduos após serem vacinados. Esta síndrome recebeu o nome de Trombocitopenia trombótica induzida por vacina (VITT – *vaccine-induced immune thrombotic thrombocytopenia*). Duas vacinas que utilizam vetores de adenovírus foram implicadas em causar VITT:

- ChAdOx1 nCoV-19 (AstraZeneca, Universidade de Oxford e Serum Institute of India);
- Ad26.COV2.S (Janssen; Johnson & Johnson).

Embora seja reconhecida como uma reação adversa a essas vacinas, a **incidência** real de VITT é ainda desconhecida, e evidências apontam para uma complicação rara. A maioria dos relatos descreve um pequeno número de casos entre dezenas de milhões de indivíduos vacinados.^1-4^ Em janeiro de 2022, um relatório do sistema de notificação norte-americano de eventos adversos da vacina (VAERS, *Vaccine Adverse Event Reporting System*) identificou 54 casos de trombose entre mais de 14 milhões de receptores de Ad26.COV2.S, uma incidência de 3,8 por milhão (aproximadamente 1 em 263.000).^2,3^ A farmacovigilância para esses desfechos tem sido minuciosa, sugerindo que a apuração de casos tem alta confiabilidade.

Os **fatores de risco** para VITT são desconhecidos. Sexo feminino, presença de obesidade e idade entre 30 e 50 anos foram propostos como fatores de risco com base nos relatórios iniciais, mas podem refletir apenas a demografia das populações vacinadas precocemente.^1,3-5^ Nos EUA, o risco de VIITT após administração de Ad26.COV2.S foi estimada em 3,8 casos por milhão de doses na população em geral, e 9 a 10,6 casos e por milhão de doses para mulheres de 30 a 49 anos.^2,3,6^

Embora a trombose seja a **apresentação clínica** na maioria dos casos relatados,^1,4,7^plaquetopenia isolada também pode ocorrer.^7,8^ A trombose da veia cerebral é uma das formas mais comuns de acometimento descritas.

O **prognóstico** da VITT depende do território acometido, extensão da trombose e complicações decorrentes, e tempo para o diagnóstico. Em uma série de 220 indivíduos com VITT definitiva ou provável, a taxa de mortalidade foi de 22%.^5^ Os fatores identificados que conferem maior risco de morte incluem trombose venosa cerebral, trombocitopenia mais acentuada e complicações hemorrágicas concomitantes. Nos EUA, a mortalidade relacionada à VITT foi de 0,57 mortes por milhão de doses de Ad26.COV2.S no geral, e de 1,8 a 1,9 mortes por milhão de doses em mulheres de 30 a 49 anos.^2,3^ Comparativamente, a taxa de mortalidade global por COVID-19 é de 1 a 2%. A incidência de trombose chega a 8% de todos os pacientes hospitalizados com COVID-19, e até 23% em indivíduos em unidades de terapia intensiva.^9^ Ainda, há evidências de que a incidência de trombose da veia cerebral em pacientes hospitalizados com COVID-19 foi de 207 por milhão de casos, muito superior à incidência de casos induzidos por vacina (0,9 a 3,8 por milhão).^10^

Assim, existe um consenso de que os benefícios da vacinação **superam os riscos potenciais** de efeitos colaterais raros da vacina, como VITT.^11^

Recomendações específicas:

- Uma **história prévia de tromboembolismo venoso** (TEV) ou uma predisposição para TEV **não são uma contraindicação** à vacinação com qualquer tipo de vacina. Nenhum estudo demonstrou um risco aumentado de VITT ou outras complicações trombóticas após vacinação nestes indivíduos;
- Para indivíduos que receberam uma primeira dose da vacina ChAdOx1 nCoV-19 e **não desenvolveram VITT**, a recomendação é completar o esquema vacinal com duas doses. Não há evidências de que a segunda dose, ou mesmo o reforço (*booster*), aumente o risco de complicações trombóticas. Uma revisão do banco de dados de segurança da AstraZeneca na Europa e no Reino Unido identificou uma incidência de 8,1 casos de VITT por um milhão de primeiras doses, e apenas 2,3 casos por milhão de segundas doses;^12^
- Para **indivíduos que tiveram VITT **com uma vacina de vetor de adenovírus, outra dose não deve ser administrada. Recomenda-se fazer a transição do esquema vacinal para uma vacina de RNAm;
- As evidências disponíveis **não** apoiam nenhuma avaliação clínica, laboratorial ou de imagem em **indivíduos assintomáticos** antes ou após a vacinação.^13^

### Miocardite induzida por vacina

A associação entre miocardite e vacinas é descrita como um evento adverso raro, cuja incidência é observada mais frequentemente após vacinação contra varíola, influenza e hepatite B. Entre 1990 e 2018, apenas 0,1% das mais de 620 mil notificações de reações adversas pós-vacinas foram atribuíveis à miopericardite nos EUA.^14^

Em julho de 2021, os Centros de Controle e Prevenção de Doenças (CDC) relatou uma possível associação entre as vacinas de RNAm para SARS-CoV-2 e casos de miocardite e pericardite. As duas vacinas que utilizam a tecnologia de RNAm e foram associadas a miocardite são:

- BNT162b2 (Pfizer);
- mRNA-1273 (Moderna).

Inicialmente, foi estimada uma taxa de 32,4 casos por milhão de doses aplicadas, chegando até a 66,7 por milhão após a segunda dose entre homens de 12 e 17 anos. A incidência diminuía significativamente com o aumento da idade e era marcadamente mais baixa entre mulheres de todas as idades.^15^

Após relatos iniciais de miopericardite nesse grupo etário (de 12 a 17 anos), houve uma maior atenção nas políticas de segurança e vigilância para este efeito adverso. Nos últimos meses, estudos de base populacional foram publicados sobre a ocorrência desse evento pós-vacinação para SARS-CoV-2, descritos a seguir e resumidos na Tabela 1.

**Tabela 1:** – Características dos estudos populacionais avaliando miocardite ou miopericardite associada às vacinas contra COVID-19 que utilizam RNAm

Autores	País	População do Estudo	Indivíduos vacinados com vacina RNAm	Casos de miocardite confirmada	Evento/ 100.000 vacinados (taxa bruta)	Óbitos	Vacina BTN162b2 (Pfizer)	Vacina mRNA1273 (Moderna)
Witberg et al.^16^	Israel	Banco de dados de serviço de saúde de Israel, usuários com idade ≥ 16 anos	2.558.421	54	2,1	1	100%	
Mevorach et al.^17^	Israel	Banco de dados do Ministério da Saúde de Israel, população com idade ≥ 16 anos	5.125.635	136	2,4	1	100%	
Husby et al.^19^	Dinamarca	Coorte populacional, idade ≥ 12 anos	3.981.109	69	1,7	0	87%	13%
Chua et al.^20^	Hong Kong	Dados populacionais que incluiu chineses de 12 a 17 anos	178.163	33	18,5	0	100%	
Patone et al.^21^	Grã-Bretanha	Dados do English National Immunisation (NIMS) Database, população ≥ 16 anos	17.999.580	169	0,9	25*	94%	6%
Oster et al.^23^	EUA	Registro de vigilância Vaccine Adverse Event Reporting System (VAERS), idade ≥ 12 anos	192.405.448	1626	0,85	0	70%	30%
Simone et al.^24^	EUA	Dados do Kaiser Permanente Southern California – idade ≥ 18 anos	2.392.924	15	0,58	0	50%	50%
Montgomery et al.^26^	EUA	Dados do US Military Health Service, idade entre 20 e 51 anos	2.810.000	23	0,8	0	Não disponível	

* Qualquer morte registrada tendo "miocardite" entre as causas, nos primeiros 28 dias após a primeira ou segunda dose da vacina.

- Witberg et al.^16^ identificaram 54 casos confirmados de miocardite, de acordo com os critérios do CDC, entre mais de 2,5 milhões de indivíduos vacinados e monitorados por uma organização de saúde de Israel. Entre os pacientes com miocardite, 37 (69%) foram diagnosticados entre três e cinco dias após a segunda dose da vacina. A incidência geral estimada de miocardite (aferida até 42 dias após a primeira dose de vacina) foi de 2,13 casos por 100.000 pessoas. Essas taxas foram maiores entre homens de 16 e 29 anos, 10,7 casos por 100.000 pessoas. A maioria dos casos de miocardite foi classificada como leve (76%) ou moderada (22%), e houve um caso associado a choque cardiogênico. Aqueles que apresentavam disfunção ventricular esquerda na admissão (29%) apresentaram recuperação da função ventricular no seguimento (mediana de 83 dias);
- Mevorach et al.^17^ relataram 136 casos de miocardite, definidos segundo critérios da *Brighton* *Collaboration *e do CDC, entre 5,1 milhões de indivíduos vacinados com duas doses de BNT162b2 mRNA (Pfizer) em Israel. Desses, 117 (86%) ocorreram após a segunda dose, e 81% deles foram internados nos primeiros sete dias após a vacinação. A incidência geral foi de 0,35 casos por 100 000 pessoas nos primeiros 21 dias após a primeira dose, e 2,10 casos por 100.000 pessoas após a segunda dose. A incidência entre homens foi maior, chegando a 0,6 casos por 100 000 pessoas após a primeira dose, e 3,8 casos por 100 000 pessoas após segunda dose. A incidência aumentou para 1,3 e 15,1 por 100.000 pessoas após a primeira e a segunda dose, respectivamente, entre adolescentes do sexo masculino de 16 a 19 anos. A razão da comparação da incidência de miocardite entre vacinados e não vacinados após a segunda dose foi de 2,4 (IC 95% 1,1 a 5,0). Entre esses casos identificados, 95% tiveram um curso benigno e autolimitado, e um óbito ocorreu. Recentemente, os mesmos autores investigaram os casos de hospitalizações por miocardite entre adolescentes de 12 a 15 anos e encontraram 13 casos que poderiam ser relacionados à vacina pelo critério temporal.^18^ O risco de miocardite entre adolescentes do sexo masculino foi de 0,56 casos por 100 000 após a primeira dose e 8,09 casos por 100 000 após a segunda dose, e entre as mulheres da mesma idade, 0 casos por 100 000 após a primeira dose e 0,69 casos por 100.000 após a segunda dose;
- Utilizando dados do sistema nacional de monitoramento de saúde da Dinamarca, Husby et al.^19^ acompanharam 4,9 milhões de indivíduos acima de 12 anos, entre outubro de 2020 e outubro de 2021, e observaram 269 novos casos de miocardite no período. Entre os 3 482 295 indivíduos vacinados com BNT162b2 (Pfizer), 48 desenvolveram miocardite ou miopericardite nos primeiros 28 dias após a vacina. A incidência absoluta foi de 1,4 por 100,000 vacinados. O risco de miocardite não foi significativamente diferente entre vacinados e não vacinados na análise primária de 28 dias após a vacinação [*hazard ratio* (HR) ajustada 1,34, intervalo de confiança de 95% (IC95%) 0,90 a 2,00], mas foi significativamente maior quando essa foi feita dentro de um período mais curto, 14 dias pós-exposição (HR 1,89, IC95% 1,23 a 2,90). Diferentemente de outras coortes, o risco de miocardite foi maior entre mulheres do que homens. Entre os 498.814 indivíduos que receberam mRNA-1273 (Moderna), 21 desenvolveram miocardite ou miopericardite nos primeiros 28 dias (incidência 4,2 por 100.000 indivíduos vacinados; HR 3,92, IC95% 2,30 a 6,68). A taxa de risco ajustada na população entre 12 e 39 anos foi de 1,48 (IC95% 0,74 a 2,98) com BNT162b2, e 5,24 (IC95% 2,47 a 11,12) com mRNA-1273. Houve apenas uma morte, e a apresentação clínica, em geral, foi similar aos casos de miocardite ou miopericardite entre não vacinados;
- Em Hong Kong, Chua et al.^20^ reportaram 33 casos de miocardite e/ou pericardite entre 178.163 adolescentes de 12 a 17 anos vacinados com BNT162b2 (Pfizer). Destes casos, 29 (87,9%) eram do sexo masculino, e a maioria (81,8%) desenvolveu miocardite/pericardite aguda após a segunda dose. A incidência geral foi de 18,5 por 100 000 adolescentes vacinados. Entre os jovens do sexo masculino, a incidência após a primeira e a segunda dose foi 5,57 e 37,32 por 100 000 pessoas vacinadas, respectivamente. Todos os casos foram leves e se recuperaram espontaneamente;
- Patone et al.^21^ avaliaram os riscos de miocardite, pericardite e arritmias cardíacas associadas à vacinação contra COVID-19 *versus* infecção por SARS-Cov2. Eles avaliaram mais de 38,6 milhões de adultos ingleses, e observaram que 0,001% dos indivíduos tiveram miocardite nos primeiros 28 dias após qualquer dose de vacina. Neste período, houve um caso excedente de miocardite para vacina BNT162b2 (Pfizer), e seis casos excedentes com mRNA-1273 (Moderna) para cada um milhão de indivíduos vacinados. Mesmo este risco aumentado em 10 casos para cada um milhão de vacinados após uma segunda dose de mRNA-1273, essa taxa ainda foi menor do que os 40 casos de miocardite para cada 1 milhão de indivíduos com teste positivo para SARS-Cov2. Os mesmos autores ampliaram a análise para 42 milhões de britânicos vacinados [dados em pré-publicação (*preprint*)^22^], mostrando resultados semelhantes com a dose de reforço das vacinas. Em homens com idade <40 anos, os riscos de miocardite relacionada à infecção por SARS-CoV-2 e à vacinação foram semelhantes, exceto com a vacina mRNA-1273, que ofereceu maior risco de miocardite do que aquele relacionado à infecção pelo SARS-CoV-2;
- Mais recentemente, em uma análise detalhada de casos de miocardite nos EUA, Oster et al.^23^ concluíram que o risco de miocardite após vacinas de RNAm foi maior após a segunda dose entre adolescentes e jovens do sexo masculino. Entre 192 405 448 americanos que receberam um total de 354 100 845 doses de vacina contra COVID-19 com base em RNAm, foram identificados 1 626 casos de miocardite. A maioria dos casos (82%) foi em indivíduos do sexo masculino. Com relação à BNT162b2 (Pfizer), a incidência foi de 70,6 por cada milhão de doses aplicadas entre homens de 12 a 15 anos; 105,9 por milhão em homens de 16 a 17 anos, e 52,4 por milhão de doses aplicadas em homens de 18 a 24 anos. A maioria dos casos foi considerada leve a moderada, com boa evolução. Até a publicação, havia duas notificações de óbitos potencialmente devido à miocardite que ainda estavam sob investigação.

Outros estudos, também nos EUA, já haviam relatado uma incidência de 0,58 para cada 100.000 casos num período de observação de 10 dias após a segunda dose de ambas as vacinas de RNAm;^24^ e uma estimativa de 6,3 casos excedentes de miocardite para cada um milhão de doses aplicadas, nas primeiras 3 semanas após vacinação com RNAm, em indivíduos entre 12 e 39 anos.^25^ Em militares norte-americanos, a taxa foi de 0,8 para cada 100.000 doses aplicadas, todas em homens.^26^

A comparação direta dos estudos acima apresenta limitação, pois cada um traz uma peculiaridade, quer seja nos critérios diagnósticos, período considerado, diferentes faixas etárias incluídas, além de diferenças metodológicas no cálculo do risco (absoluto ou em excesso). Ainda, em muitas análises, há uma variável que não foi estudada, que é a preexistência de miocardite, quer seja por COVID-19 ou por outras causas. Ainda assim, o conjunto de evidências sugere que o risco de miocardite aguda associada à vacinação para COVID-19 é real e tem incidência muito baixa, e é mais comumente relatada em jovens do sexo masculino.

Os **mecanismos fisiopatológicos** da inflamação e injúria miocárdicas observadas com vacinas de RNAm contra COVID-19 não estão bem estabelecidos, mas podem estar relacionados à sequência genética que codifica a proteína spike do vírus SARS-CoV-2 ou à resposta imune (reações de hipersensibilidade, por exemplo) a essas vacinas. O fato de as maiores taxas serem observadas em jovens do sexo masculino, e principalmente após a segunda dose, apoiam a hipótese de uma resposta imune mal adaptada, que pode ter influência dos hormônios sexuais.

A **apresentação clínica** é similar a um quadro clássico de miocardite aguda, e inclui dor torácica, acompanhado de dispneia ou falta de ar. Além disso, a troponina está elevada em quase a totalidade dos casos, e aproximadamente 70% possuem alguma alteração ao eletrocardiograma. Disfunção sistólica aguda, com queda na fração de ejeção do ventrículo esquerdo, foi reportada em 6 a 12% dos casos.^20,23,27,28^

O prognóstico da miocardite relacionada à vacina é muito favorável: na maioria dos casos, é autolimitada, com resolução dos sintomas e normalização dos exames laboratoriais e eletrocardiograma/ecocardiograma ao longo do seguimento. Na maior revisão de casos publicada, Kohli et al.^27^ relataram que as complicações graves com risco de vida devido permanecem extremamente raros.

Por outro lado, é preciso colocar sempre em perspectiva a magnitude dos benefícios da vacinação para toda população, quando se avalia os potenciais eventos adversos relacionados. No estudo britânico já citado, a taxa de miocardite associada à infeção pelo SARS-Cov2 com 28 dias de exposição foi de 30 casos por milhão para a população em geral, e este número chegava a 73 casos por milhão em homens acima de 40 anos.^22^ Ou seja, **a taxa de miocardite associada à COVID-19 excede a taxa observada com as vacinas na maioria dos levantamentos populacionais. A exceção ainda são homens mais jovens, particularmente adolescentes, nos quais o risco de miocardite associada às vacinas excede a taxa de miocardite por COVID-19 na mesma faixa etária.**^21,22^** Ainda assim, quando comparadas às taxas de mortalidade da infecção pelo vírus SARS-Cov2 (0,1 a 1,0 por 100.000 indivíduos entre 12 e 29 anos), bem como o risco de hospitalização, o benefício geral da vacina supera o risco de miocardite por ela induzida. **

Importante ressaltar que os indivíduos infectados pelo Sars-Cov2 têm risco aumentado para outras condições cardiovasculares que não só aquelas descritas como eventos adversos da vacinação (trombose e miocardite ou miopericardite). Abbasi et al.^29^ estimaram a ocorrência extra de eventos atribuídos a COVID-19 em um período de 12 meses comparado a grupo de controle (que não tiveram COVID-19). Para cada 1 000 pessoas, COVID-19 foi associada a um extra de: 45 casos de qualquer evento cardiovascular, 23 casos de eventos cardiovasculares maiores (infarto do miocárdio, acidente vascular cerebral e mortalidade por todas as causas), 20 casos de arritmias e 11 casos de fibrilação atrial, 12 casos de insuficiência cardíaca, 10 casos de evento tromboembólicos (5,5 embolias pulmonares e 4 tromboses venosas profundas), 7 casos de doença cardíaca isquêmica (5,3 síndromes coronarianas agudas, 3 infartos do miocárdio e 2,5 anginas), 4 casos de acidente vascular cerebral, 1,23 casos de doença inflamatória cardíaca ou pericárdica.^29^

Analisando dados epidemiológicos, Gargano et al.^30^ concluíram que os benefícios proporcionados pela vacinação (prevenir a doença provocada pelo SARS-Cov2, ou hospitalizações, internações em UTI e óbitos associados) superaram os riscos de miocardite decorrente da vacina em todas as populações para as quais a vacinação foi recomendada. O equilíbrio risco *versus* benefício variou com a idade e sexo. Para cada um milhão de homens entre 12 e 29 anos que receberam uma segunda dose de vacina de RNAm, poderiam ser evitados 11 000 casos de COVID-19, 560 hospitalizações, 138 internações em UTI, e seis mortes, em comparação com 39 a 47 casos esperados de miocardite nessa mesma população.^31^

Ainda, evidências mostram que a vacinação com a BNT162b2 (Pfizer) tem eficácia de 91% contra Síndrome Inflamatória Multissistêmica em Crianças (MIS-C) em adolescentes com idades entre 12 e 18 anos, e protege contra os cursos clínicos mais graves da síndrome.^32^ MIS-C é observada a uma taxa de 316 por 1 milhão de casos de SARS-CoV-2,^33^ com predomínio do sexo masculino,^34^ e frequentemente resulta em internações prolongadas e necessidade de cuidados intensivos, ao contrário da maioria dos casos leves de miocardite associada à vacina.

Por fim, mesmo numa fase de escalada da variante Ômicron, quando novos questionamentos podem surgir sobre os riscos *versus* benefícios da vacinação em crianças e adolescentes jovens, uma série de estudos publicados mais recentemente concluíram que a vacinação com RNAm (particularmente BNT162b2/Pfizer), especialmente quando reforçada com uma terceira dose, permanece altamente eficaz contra formas graves da COVID-19, incluindo morte.^35^ Ainda, a terceira dose da vacina confere eficácia de 82% em evitar atendimentos de urgência e emergência, e 90% em prevenir hospitalizações.^36^ Ou seja, as doses de reforço ainda oferecem proteção adicional contra formas graves tanto das variantes Ômicron quando Delta,^30^ que, não podemos negligenciar, ainda circula em nosso meio. A Tabela 1 descreve as características dos estudos populacionais avaliando miocardite ou miopericardite associada às vacinas contra COVID-19 que utilizam RNAm.

### Manejo da suspeita de miocardite ou miopericardite associada à vacina

A suspeita de miocardite ou miopericardite deve ser considerada em pacientes vacinados com a BNT162b2 (Pfizer) ou a mRNA-1273 (Moderna) que apresentem sintomas de dor ou desconforto torácico (sintoma predominante), dispneia ou taquipneia, fadiga, palpitações, síncope, inapetência, letargia e tenham sido submetidos a eletrocardiograma, ecocardiograma, dosagem de troponina e ressonância magnética nuclear, excluindo-se a suspeita de outra causa.^37^Em nenhum dos estudos, foram feitas análises ou revisões comparando os tipos de tratamento administrado, e em quase todos o tratamento foi conservador. Além dos cuidados gerais, a maioria dos pacientes recebeu ibuprofeno, alguns receberam corticosteroides, e uma minoria corticoide e imunoglobulina. Para pacientes com disfunção sistólica induzida pela miocardite, podemos inferir que foi empregada terapia usual com betabloqueadores, inibidores da enzima de conversão de angiotensina ou um bloqueador de angiotensina II ou o sacubitril/valsartan, mais um antagonista de receptor de mineralocorticoide, e talvez um inibidor de SGLT2.^23,24,29,38^

## Conclusões

As vacinas contra COVID-19 são seguras e seus benefícios superam em larga escala os riscos de efeitos adversos relacionados. Os principais efeitos adversos cardiovasculares associados a essas vacinas são a VITT e a miocardite. Ao passo em que o primeiro está associado às vacinas que utilizam vetor de adenovírus, o segundo é observado entre as vacinas com tecnologia de RNAm.

Miocardite associada à vacina permanece um evento adverso raro, embora a incidência entre adolescentes do sexo masculino possa chegar até 107 casos por milhão de doses, e excede a incidência de miocardite associada à COVID-19 na mesma parcela da população.

No entanto, como o curso clínico da miocardite associada à vacina é geralmente leve e autolimitada, mesmo entre os adolescentes do sexo masculino, a totalidade do efeito protetor da vacinação contra COVID-19, particularmente na prevenção de COVID-19 grave, hospitalização, MIS-C e morte, continua a exceder claramente o risco de miocardite induzida.

Na faixa etária pediátrica, os benefícios vão além daqueles diretamente relacionados à saúde do próprio paciente, também diminuindo a transmissão da COVID-19 nessa faixa etária e, de forma indireta, para indivíduos mais velhos. A vacinação reduz a necessidade de medidas de mitigação nas escolas, minimizando as interrupções na educação das crianças e a manutenção de seu bem-estar geral, saúde e segurança.
