# Supplementary material for: Posicionamento sobre Segurança Cardiovascular das Vacinas contra COVID-19 - 2022
Source: Arq Bras Cardiol. 2022 Apr 7;118(4):789–96. [Article in Portuguese] doi: 10.36660/abc.20220179 (PMC9007012; doi:10.36660/abc.20220179)
Supplement: Translation

# Translation: Position Statement on Cardiovascular Safety of Vaccines Against COVID-19 - 2022

**Authors:** Humberto Graner Moreira, Múcio Tavares de Oliveira, Bruno Pereira Valdigem, Cristiane Nunes Martins, Carisi Anne Polanczyk

**Affiliations:** 1 Brazil Liga de Hipertensão Arterial - Universidade Federal de Goiás, Goiânia, GO – Brazil; 2 Brazil Hospital Israelita Albert Einstein, São Paulo, SP – Brazil; 3 Brazil Centro de Infusão e Hospital Dia do InCor, São Paulo, SP – Brazil; 4 Brazil Faculdade de Medicina da Universidade de São Paulo (FMUSP), São Paulo, SP – Brazil; 5 Brazil Departamento de Insuficiência Cardíaca da Sociedade Brasileira de Cardiologia (DEIC) - Diretoria 2022-2023, Rio de Janeiro, RJ – Brazil; 6 Brazil Projeto Insuficiência Cardíaca da Sociedade de Cardiologia do Estado de São Paulo (SOCESP), São Paulo, SP – Brazil; 7 Brazil Rede D’Or, Rio de Janeiro, RJ – Brazil; 8 Brazil Instituto Dante Pazzanese de Cardiologia, São Paulo, SP – Brazil; 9 Brazil Biocor Instituto, Nova Lima, MG – Brazil; 10 Brazil Programa de Pós-graduação em Cardiologia da Universidade Federal do Rio Grande do Sul, Porto Alegre, RS – Brazil; 11 Brazil Hospital de Clínicas de Porto Alegre, Porto Alegre, RS – Brazil; 12 Brazil Instituto Nacional de Avaliação de Tecnologia em Saúde, Porto Alegre, RS – Brazil; 13 Brazil Hospital Moinhos de Vento, Porto Alegre, RS – Brazil

**Table:** 

Position Statement on Cardiovascular Safety of Vaccines Against COVID-19 – 2022	
The report below lists declarations of interest as reported to the SBC by the experts during the period of the development of these statement, 2022.	
Expert	Type of relationship with industry
Bruno Pereira Valdigem	Nothing to be declared
Carisi Anne Polanczyk	Financial declaration A - Economically relevant payments of any kind made to (i) you, (ii) your spouse/partner or any other person living with you, (iii) any legal person in which any of these is either a direct or indirect controlling owner, business partner, shareholder or participant; any payments received for lectures, lessons, training instruction, compensation, fees paid for participation in advisory boards, investigative boards or other committees, etc. From the brazilian or international pharmaceutical, orthosis, prosthesis, equipment and implants industry: - Bayer, Pfizer, Novartis, Roche, Amgen, Bristol. Other relationships Funding of continuing medical education activities, including travel, accommodation and registration in conferences and courses, from the brazilian or international pharmaceutical, orthosis, prosthesis, equipment and implants industry: - Bayer, Roche.
Cristiane Nunes Martins	Nothing to be declared
Humberto Graner Moreira	Financial declaration A - Economically relevant payments of any kind made to (i) you, (ii) your spouse/partner or any other person living with you, (iii) any legal person in which any of these is either a direct or indirect controlling owner, business partner, shareholder or participant; any payments received for lectures, lessons, training instruction, compensation, fees paid for participation in advisory boards, investigative boards or other committees, etc. From the brazilian or international pharmaceutical, orthosis, prosthesis, equipment and implants industry: - Novartis: Entresto; Bayer: Xarelto; Pfizer: Eliquis; Libbs: Plenance Eze.
Mucio Tavares de Oliveira Júnior	Financial declaration B - Research funding under your direct/personal responsibility (directed to the department or institution) from the brazilian or international pharmaceutical, orthosis, prosthesis, equipment and implants industry: - Sanofi Pasteur: FLUZONE Senior; Torrent: experimental drug. Other relationships Funding of continuing medical education activities, including travel, accommodation and registration in conferences and courses, from the brazilian or international pharmaceutical, orthosis, prosthesis, equipment and implants industry: - Astra Zeneca, Boehringer, Novartis, Torrent Pharma, Sanofi Pasteur, Merck, Biolab.

**Central Illustration:** : Position Statement on Cardiovascular Safety of Vaccines Against COVID-19 - 2022

Arq Bras Cardiol. 2022; 118(4):789-796.

## Introduction

The Scientific Committee of the Brazilian Society of Cardiology, by determination of its Administrative Council, has convened a work group to monitor and set up scientific evidence on cardiovascular safety of vaccines against COVID-19 in a continuous and systematic way. This group aims to reproduce scientifically solid data, summarize currently available evidence, and develop recommendations to Brazilian cardiologists in the form of positions of the Brazilian Society of Cardiology.

Vaccines to prevent SARS-CoV-2 infection are considered the most effective strategy to control the pandemic. Despite the short time for vaccine development, each vaccine approved has gone through all preclinical and clinical stages (phase I and II) of clinical research.

The strict standards of safety applied to these studies are maintained during the so-called “phase IV”, or post-marketing surveillance. This stage is crucial for evaluating the occurrence of rare adverse events whose causal relationship with vaccines can only be established after they were administered to a large number of people.

Like other vaccines, adverse events, including those related to the circulatory system, have been observed during this surveillance phase of immunization programs against COVID-19. Here we review the evidence on two cadiovascular adverse effects – thrombosis with thrombocytopenia and vaccine-induced myocarditis.

### Vaccine-induced immune thrombotic thrombocytopenia (VITT)

In February 2021, a post-thrombotic syndrome was described in some vaccinated individualsand the syndrome was named vaccine-induced immune thrombotic thrombocytopenia (VITT). Two adenovirus-vectored vaccines have been implicated in the cause of VITT:

- ChAdOx1S nCoV-19 (Oxford/AstraZeneca and Serum Institute of India);
- Ad26.COV2.S (Janssen; Johnson & Johnson)

Although recognized as a vaccine adverse event, the real **incidence** of VITT is still unknown, and evidence has suggested it as a rare complication. Most reports have described a small number of cases among tens of millions of vaccinated people.^1-4^ In January 2022, a report of the Vaccine Adverse Event Reporting System (VAERS) identified 54 cases of thrombosis among more than 14 million Ad26.COV2.S recipients, an incidence of 3.83 per million (approximately 1 in 263,000).^2,3^Close surveillance of these outcomes has been made, and suggested high reliability of the reports.

The **risk factors** for VITT are still unknown. Female sex, obesity, and age between 30 and 50 years have been proposed as risk factors based on initial reports, although they may merely reflect the demography of early-vaccinated populations.^1,3-5^ In the United States, the risk of VITT after Ad26.COV2.S vaccination was estimated at 3.8 cases per million of doses in the general population, and between nine and 10.6 cases per million of doses for women aged between 30 and 49 years.^2,3,6^

Although thrombosis is the most common **clinical presentation**,^1,4,7^thrombocytopenia alone may also occur.^7,8^ Cerebral venous thrombosis is one of the most commonly described.

The **prognosis** of VITT depends on the site, extension and complication of thrombosis, and time for diagnosis. In a series of 220 individuals with definite or probable VITT, a mortality rate of 22% was reported.^5^ Factors associated with increased risk of death include cerebral venous thrombosis, severe thrombocytopenia and concomitant bleeding complications. In the US, VITT-related mortality was 0.57 deaths per million doses of Ad26.COV2.S in total population, and 1.8-1.9 deaths per million doses among women aged between 30 and 49 years.^2,3^ Comparatively, the overall mortality rate of COVID-19 is 1-2%. The incidence of thrombosis reaches 8% of all patients hospitalized for COVID-19, and 23% of intensive care unit patients.^9^ In addition, there is evidence that the incidence of cerebral venous thrombosis in patients hospitalized for COVID-19 was 207 per million cases, much higher than the incidence of vaccine-induced thrombosis (0.9-3.8 per million cases).^10^

Therefore, there is a consensus that the benefits of vaccination **surpass the potential ri**sks of rare side effects of the vaccine, like VITT.^11^

### Specific recommendations:

- Previous **history of venous thromboembolism** (VTE) or predisposition for VTE **are not contraindications** for COVID-19 vaccination, regardless of the type of vaccine. No study has shown an increased risk of VITT or other thrombotic complications after vaccination in these individuals;
- Individuals who received the first dose of ChAdOx1 nCoV-19 and **did not develop VITT** should complete the vaccination schedule of two doses. There is no evidence that the second dose (or even the booster) increases the risk of thrombotic complications. A review of AstraZeneca safety database in Europe and United Kingdom identified an incidence of 8.1 cases of VITT per million for the first doses, and of only 2.3 cases per million for the second doses;^12^
- Individuals who received an adenovirus-vectored vaccine and developed VITT should not receive a second dose. A transition to a mRNA vaccine schedule is recommended;
- Available evidence **does not** support the performance of any clinical, laboratory or imaging tests in **asymptomatic individuals** before or after vaccination.^13^

### Vaccine-induced myocarditis

The association between myocarditis and vaccines has been described as a rare adverse event. Its incidence has been more commonly reported in smallpox, influenza, and hepatitis B vaccines. From 1990 to 2018, only 0.1% of more than 620 thousand notifications of post-vaccine adverse events were attributed to myopericarditis in the United States.^14^

In July 2021, the Centers for Disease Control and Prevention (CDC) reported a possible association between mRNA vaccines and SARS-CoV-2 in myocarditis and pericarditis. The two mRNA-based vaccines that have been associated with myocarditis are:

- BNT162b2 from Pfizer
- mRNA-1273 from Moderna

First, it was estimated an incidence of 32.4 cases per million doses, 66.7 cases per million doses in males aged 12–17 years following the second dose. The incidence significantly decreased with age and was markedly lower among women of all ages.^15^

After initial reports of myopericarditis in this age group (12-17 years old), greater attention has been paid to politics of safety and surveillance of this adverse event. In recent months, population-based studies on the occurrence of this event after vaccination against SARS-CoV-2 have been published. These studies will be described below and are summarized in Table 1.

**Table 1:** – Characteristics of population-based studies evaluating myocarditis or myopericarditis associated with mRNA vaccines against COVID-19

Authors	Country	Study population	Individuals vaccinated with mRNA vaccine	Number of cases of confirmed myocarditis	Event/ 100,000 vaccinated (absolute rate)	Deaths	BTN162b2 vaccine (Pfizer)	mRNA1273 vaccine (Moderna)
Witberg et al.^16^	Israel	Database of the health care organization in Israel, of members ≥ 16 years old	2,558,421	54	2.1	1	100%	
Mevorach et al.^17^	Israel	Database of the Israeli Ministry of Health, people ≥ 16 years old	5,125,635	136	2.4	1	100%	
Husby et al.^19^	Denmark	Population cohort of people ≥ 12 years old	3,981,109	69	1.7	0	87%	13%
Chua et al.^20^	Hong Kong	Population cohort including adolescents aged between 12 and 17 years	178,163	33	18.5	0	100%	
Patone et al.^21^	Great-Britain	Data from the English National Immunisation (NIMS) Database, people aged ≥ 16 years	17,999,580	169	0.9	25*	94%	6%
Oster et al.^23^	USA	Vaccine Adverse Event Reporting System (VAERS), people ≥ 12 years old	192,405,448	1626	0.85	0	70%	30%
Simone et al.^24^	USA	Data from the Kaiser Permanente Southern California – members ≥ 18 years old	2,392,924	15	0.58	0	50%	50%
Montgomery et al.^26^	USA	Data from the US Military Health Service, member aged between 20 and 51 years	2,810,000	23	0.8	0	Not available	

* Any death caused by myocarditis (among other causes) registered within 28 days after the first or the second dose of the vaccine.

- Witberg et al.^16^ identified 54 cases that met the CDC criteria for myocarditis among more than 2.5 million vaccinated individuals, who were monitored by a health organization in Israel. Among the patients with myocarditis, 37 (69%) received the diagnosis between three and five days after the second vaccine dose. The estimated incidence of myocarditis (measured within 42 days after the first dose of the vaccine) was 2.13 cases per 100,000 persons. The highest incidence of myocarditis was reported in male patients aged between 16 and 29 years (10.7 cases per 100,000 persons). Most cases of myocarditis were described as mild (76%) or intermediate (22%) as intermediate; one case was associated with cardiogenic shock. Patients who had left ventricular dysfunction on echocardiography during admission (29%) had normal ventricular function after a median follow-up of 83 days.
- Mevorach et al.^17^ reported 136 cases of myocarditis, defined according to the Brighton Collaboration and the CDC criteria among 5.1 million individuals vaccinated with two doses of BNT162b2 mRNA (Pfizer) in Israel. Of these, 117 (85%) presented myocarditis after the second dose, and 81% were hospitalized within seven days after vaccination. The incidence ratio was 0.35 cases per 100,000 within 21 days after the first dose, and 2.10 cases per 100,000 individuals after the second dose. The incidence increased from 1.3 to 15.1 per 100,000 individuals after the first and second dose, respectively, among male teenagers aged between 16 and 19 years. The ratio for the comparison of the incidence of myocarditis between vaccinated and unvaccinated persons after the second dose was 2.35 (95% CI, 1.1 to 5.0). Most (95%) of these cases were self-restricted and had a benign course, and one death has occurred. Recently, the same authors investigated the cases of hospitalization for myocarditis in adolescents aged between 12 and 15 years and found 13 cases possibly related to the vaccine based on a temporal criterion.^18^ The risk of myocarditis among male adolescents was 0.56 cases per 100,000 after the first dose, and among female adolescents with the same age, the risk was 0 cases per 100,000 after the first dose and 0.69 cases per 100,000 after the second dose.
- Using information on vaccinations from the Danish Vaccination Register, Husby et al.^19^ followed 4.9 million residents aged over 12 years between October 2020 and October 2021, and identified 269 new cases of myocarditis in the period. Of 3,482,295 individuals vaccinated with BNT162b2 (Pfizer), 48 developed myocarditis or myopericarditis within 28 days of vaccination. The absolute incidence was 1.4 per 100,000 vaccinated individuals. The risk of myocarditis was not significantly different between vaccinated and non-vaccinated individuals within 28 days after vaccination (adjusted hazard ratio [HR] 1.34 (95% confidence interval [95%CI] 0.90 to 2.00), but was significantly higher when using a shorter analysis time (14 days post exposure) (HR 1.89; 95%CI 1.23-2.90). Unlike other cohorts, the risk of myocarditis was higher among women than men. Among 498,814 individuals vaccinated with mRNA-1273 (Moderna), 21 developed myocarditis or myopericarditis within 28 days from vaccination date (incidence of 4.2 per 100,000 vaccinated individuals; HR 3.92, 95%CI 2.30-6.68). The adjusted hazard ratio among 12-39-year-old individuals was 1.48 (95%CI0.74-2.98) with BNT162b2, and 5.24 (95%CI 2.47-11.12) with mRNA-1273. .24 (2.47 to 11.12). Only one death occurred, and clinical outcomes were generally similar between vaccinated and unvaccinated individuals;
- In Hong Kong, Chua et al.^20^ reported 33 cases of myocarditis and/or pericarditis among 178,163 adolescents from 12 to 17 years of age vaccinated with BNT162b2 (Pfizer). Twenty-nine were males and most cases (81.8%) developed acute myocarditis/pericarditis after the second dose. The overall incidence was 18.5 per 100,000 persons vaccinated. The incidence after the first and second doses were 5.57 and 37.32 per 100,000 persons vaccinated, respectively. All patients had mild diseases and recovered spontaneously;
- Patone et al.^21^ evaluated the risks of myocarditis, pericarditis and arrhythmias associated with COVID-19 vaccination *versus* SARS-CoV2 infection. The authors evaluated more than 38.6 million adults in England and observed that 0.001% of individuals had myocarditis in the 28 days following the first or the second dose of the vaccine. In this period, there was one extra myocarditis event per million people vaccinated with BNT162b2 (Pfizer), and six extra myocarditis events per one million with mRNA-1273 (Moderna). Even the extra 10 extra myocarditis events per one million people vaccinated after a second dose of mRNA-1273 would be lower than the extra 40 myocarditis events per one million patients following a SARS-CoV-2 positive test. The same authors expanded their study to include 42 million vaccinated people in England (preprint data),^22^ showing similar results following a booster of the vaccines. In men younger than 40 years, similar findings were obtained regarding the risk of myocarditis related to SARS-CoV-2 infection and vaccination, except for the mRNA-1273 vaccine, that posed a higher risk than that related to SARS-CoV-2 infection;
- More recently, in a detailed analysis of cases of myocarditis in the United States, Oster et al.^23^ concluded that the risk of myocarditis following mRNA-based vaccines was higher after the second dose in adolescents and young male adults. Among 192 405 448 persons receiving a total of 354 100 845 mRNA-based COVID-19 vaccines, there were 1,626 cases of myocarditis, mostly (82%) in males. Regarding the BNT162b2 vaccine (Pfizer), the incidence of myocarditis was 70.6 per million doses in adolescent males aged 12 to 15 years, 105.9 per million doses in adolescent males aged 16 to 17 years, and 52.4 per million doses in young men aged 18 to 24 years. Most cases were mild or moderate, with a favorable course. Until publication, the were two death notifications potentially duet to myocarditis, still under investigation.

Other studies, also carried out in the United States, have reported an incidence of 0.58 cases per 100,000 within 10 days following the second dose of both mRNA vaccines,^24^ and an estimate of 6.3 extra myocarditis event per one million of doses administered in the first three weeks of vaccination with mRNA vaccine in individuals between 12 and 39 years of age.^25^ In members of the US Military, the incidence of myocarditis was 0.8 per 100,000 of doses administered among male military members.^26^

The direct comparison of these studies has limitations, due to peculiarities of each study, including differences in diagnostic criteria, study period, age range of populations, methods for risk calculation (absolute or excess risk). In addition, one variable has not been included in several analyses, which is the pre-existence of myocarditis, be it for COVID-19 or due to other causes. Altogether, the available evidence suggests that the risk of acute myocarditis associated with COVID-19 vaccination is real but has a very low incidence and is more commonly reported in young men.

The **pathophysiological mechanisms** of myocardial inflammation and injury described in mRNA vaccines against COVID-19 have not been well established, and they may be related to gene sequence that encodes the SARS-CoV-2 spike protein or to the immune response (e.g. hypersensitivity reactions) to these vaccines. The fact that the highest rates have been observed in young male individuals, and mainly following the second dose, supports the hypothesis of a maladaptive immune response, which may be influenced by sex hormones.

**Clinical presentation** is vaccine-induced myocarditis similar to the classical presentation of acute myocarditis and includes chest pain and dyspnea. Besides, troponin levels are increased in almost all cases, and nearly 70% have some electrocardiographic changes. Acute systolic dysfunction, with a drop in left ventricular ejection fraction, was reported in 6-12% of cases.^20,23,27,28^

The **prognosis** of vaccine-related myocarditis is very favorable; in most cases, it is self-limited, with resolution of symptoms and normalization of laboratory tests, electrocardiogram and echocardiogram over the follow-up period. In the most comprehensive review of published cases, Kohli et al.^27^ reported that serious life-threatening complications due to vaccine-related myocarditis remain rare.

On the other hand, we must always consider the magnitude of the benefits of vaccination to the whole population in the analysis of potential adverse events. In the British study mentioned above, the rate of myocarditis associated with SARS-Cov2 within 28 days of vaccination was 30 cases per million in the general population, and 73 cases per million among men older than 40 years.^22^ Thus, **based on most population-based studies currently available, the incidence of myocarditis associated with COVID-19 surpasses the incidence of myocarditis associated with vaccines. One exception is younger men, mainly adolescents, in whom the risk of vaccine-associated myocarditis exceeds that of myocarditis related to COVID-19 at the same age range.**^21,22^** Even so, as compared with mortality rates of SARS-CoV2 infection (0.1 to 1.0 per 100,000 individuals between 12 and 29 years of age), and with the risk of hospitalization, the overall benefits of vaccination overweigh the related risk of myocarditis. **

It is important to highlight that individuals infected with SARS-CoV2 are at increased risk for cardiovascular diseases, other than those recognized as vaccination adverse effects (thrombosis and myocarditis or myopericarditis). Abbasi et al. ^29^ estimated the risks and excess burden of cardiovascular outcomes attributed to COVID-19 in a 12-month-period and compared it to a control group (individuals that did not have COVID-19). For every 1,000 people, COVID-19 was associated with an extra: 45 cases of any cardiovascular event, 23 cases of major adverse cardiovascular events (myocardial infarction, stroke, and all-cause mortality), 20 cases of dysrhythmias and 11 cases of atrial fibrillation, 12 cases of heart failure, 10 cases of thromboembolic disorders (5.5 cases of pulmonary embolism and four of deep vein), seven cases of ischemic heart disease thrombosis (5.3 cases of acute coronary disease, three cases of myocardial infarction, and 2.5 incidents of angina), four cases of stroke, 1.23 cases of inflammatory disease of the heart or pericardium.^29^

Based on an analysis of epidemiological data, Gargano et al.^30^ concluded the benefits of COVID-19 vaccination (prevention of SARS-CoV2 infection, and associated hospitalizations, intensive care unit [ICU] admissions and death) outweigh the risks of myocarditis after vaccination in all populations to whom vaccination was recommended. The balance between risk and benefit varied with age and sex. Per million males aged between 12 and 29 years receiving the second dose of mRNA COVID-19 vaccine, 11,000 COVID-19 cases, 560 hospitalizations, 138 ICU admissions, and six deaths due to COVID-19 could be prevented, compared with 39-47 expected myocarditis cases in this population.^31^

Also, evidence suggests that BNT162b2 (Pfizer) shows efficacy of 91% against multisystem inflammatory syndrome (MIS) in adolescents aged 12-18 years and prevents the progression to severe stages.^32^ The incidence of MIS-C is 316 per million of SARS-CoV-2 infection cases,^33^ affecting predominantly males,^34^ frequently resulting in prolonged hospitalizations and need for intensive care, in contrast to most cases of vaccine-associated myocarditis.

Finally, even with the escalation of the Omicron variant of COVID-19, when new questions about the risks versus benefits of vaccination in children and young adolescents may arise, data from recently published studies have shown that vaccination with mRNA vaccines (particularly BNT162b2/Pfizer), especially when boosted with a third dose, remains highly effective against severe forms of COVID-19, including death.^35^ Besides, the third dose is 82% effective in preventing the need for urgency and emergency care, and 90% effective in preventing hospitalizations.^36^ Thus, a booster dose of the COVID-19 vaccine also provides additional protection against severe diseases caused by both Omicron and Delta,^30^ which are still circulating in our environment. Table 1 describes characteristics of population-based studies evaluating myocarditis or myopericarditis associated with mRNA-based vaccines against COVID-19.

### Management of suspected myocarditis or myopericarditis associated with vaccines

Myocarditis or myopericarditis should be suspected in patients vaccinated with BNT162b2 (Pfizer) or mRNA-1273 (Moderna) who present symptoms of chest pain or discomfort (predominantly), dyspnea or tachypnea, fatigue, palpitations, syncope, inappetence and lethargy, and results of electrocardiogram, echocardiography, nuclear magnetic resonance excluding other suspected causes.^37^

None of the studies performed an analysis or a review comparing the types of treatment administered, and in almost all studies treatment was conservative. In addition to general care, most patients received ibuprofen, some received corticosteroids, and a minority received corticosteroids and immunoglobulins. We can infer that patients with systolic dysfunction induced by myocarditis received traditional treatment with angiotensin converting enzyme inhibitors or an angiotensin II receptor blocker or sacubitril/valsartan, combined with a mineralocorticoid receptor antagonist (perhaps a SGLT2 inhibitor).^23,24,29,38^

## Conclusions

Vaccines against COVID-19 are safe and their benefits far outweigh the risks of associated adverse effects. The main cardiovascular adverse effects associated with these vaccines are VITT and myocarditis. While the former has been associated with adenovirus-vectored vaccines, the latter has been observed in mRNA vaccine recipients.

Vaccine-related myocarditis remains a rare adverse event, although its incidence among males can reach 107 cases per million doses, higher than myocarditis associated with COVID-19 in this same population.

However, since the course of myocarditis associated with vaccines is generally mild and self-limited, even in male adolescents, the overall protective effect of COVID-19 vaccines, particularly for the prevention of severe COVID-19, hospitalization, MIS-C and death, still overcomes the risk of vaccine-induced myocarditis.

As for children, the benefits are beyond those directly related to patient health, by decreasing the risk of direct transmission in this age group and, indirectly, to older individuals. Vaccination reduces the need of mitigation measures at schools, minimizes school interruptions and helps in the maintenance of well-being, health and safety of children.
